# Navigation by anomalous random walks on complex networks

**DOI:** 10.1038/srep37547

**Published:** 2016-11-23

**Authors:** Tongfeng Weng, Jie Zhang, Moein Khajehnejad, Michael Small, Rui Zheng, Pan Hui

**Affiliations:** 1 HKUST-DT System and Media Laboratory, Hong Kong University of Science and Technology, HongKong; 2 Centre for Computational Systems Biology, Fudan University, China; 3 The University of Western Australia, Crawley, WA 6009, Australia; 4 Mineral Resources, CSIRO, Kensington, WA, Australia

## Abstract

Anomalous random walks having long-range jumps are a critical branch of dynamical processes on networks, which can model a number of search and transport processes. However, traditional measurements based on mean first passage time are not useful as they fail to characterize the cost associated with each jump. Here we introduce a new concept of mean first traverse distance (MFTD) to characterize anomalous random walks that represents the expected traverse distance taken by walkers searching from source node to target node, and we provide a procedure for calculating the MFTD between two nodes. We use Lévy walks on networks as an example, and demonstrate that the proposed approach can unravel the interplay between diffusion dynamics of Lévy walks and the underlying network structure. Moreover, applying our framework to the famous PageRank search, we show how to inform the optimality of the PageRank search. The framework for analyzing anomalous random walks on complex networks offers a useful new paradigm to understand the dynamics of anomalous diffusion processes, and provides a unified scheme to characterize search and transport processes on networks.

Complex networks are ubiquitous in the real world ranging from sociology to biology and technology^1^. Going beyond the interesting topological properties, quantifying the impact of structural organization of networks on transport processes has become one of the most important topics. As a paradigmatic transport process, random walks on complex networks have been intensively studied^2^,^3^,^4^,^5^,^6^. A variety of measurements including mean first passage time (MFPT)^2^, first passage time^4^, and average trapping time^6^ have been proposed, providing a comprehensive characterization of random walks on networks. Moreover, these studies also facilitate our understanding of diverse dynamical processes on networks including epidemic spreading^7^, synchronization^8^, and transportation^9^.

However, for random walks, the walker is confined only to the neighbourhood of a node in each jump, which cannot model some real situations^10^, and also impedes search and transport efficiency on networks^4^. This limitation is circumvented by the natural of Lévy walks model^11^,^12^. Recently, intensive attention has been devoted to anomalous random walks on networks, such as Lévy walks^13^,^14^,^15^, traditional web surfing^16^, and even electric signals transmitted in brain networks^10^. One striking feature of anomalous random walks is having the long-range hopping (i.e., the walker can hop to far away nodes not directly connected to its current position). In fact, the occurrence of long-range hopping is frequently encountered in our life. For example, we usually communicate with people socially close to us, but also occasionally with those that are unconnected^14^. Analogously, when doing web surfing, one usually proceeds by following the hyperlinks but casually may open a new tab to look for the related topic^10^. Although it is widely agreed that anomalous random walks represent an important branch of search and transport processes on networks, how to characterize anomalous random walks and specifically, how to uncover the interplay between their dynamics and the underlying network structure has not been addressed. Traditional measurements like the mean first passage time neglect the difference between the cost associated with the nearest-neighbor jump and the long-range hopping, therefore cannot properly characterize anomalous random walks on networks.

In this paper, we propose the mean first traverse distance that represents the expected traverse distance required by a walker moving from a source node to a target node. Importantly, this allows the cost associated with hopping to be taken into account in the characterization of anomalous random walks; this therefore overcomes the problems of traditional measurements adopted in general random walks. We obtain analytically the MFTD and the global MFTD on arbitrary networks. Results on Lévy walks demonstrate that these measurements can effectively characterize the relationship between network structure and anomalous random walks. Moreover, when applied to the PageRank search, we show how to inform the optimality of the PageRank search. The new metric enables effective characterization of dynamics of anomalous random walks on networks, which promises more efficient search and transport processes on networks.

## Results

### The MFTD of anomalous random walks

We start from an undirected network consisting of *N* nodes. The connectivity of nodes is fully described by a symmetric adjacency matrix *A*, whose entry *a*_*ij*_ = 1 (0) if nodes *i* and *j* are (not) connected. For anomalous random walks, at each time step, the walker jumps from current node *i* to node *j* with a nonzero transition probability *p*_*ij*_ regardless of the connection profile of node *i*. Take Lévy walks on networks for example, the transition probability is defined as 
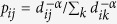
, where *α* is the tuning exponent lying in the interval 0 ≤ *α* < ∞ and *d*_*ij*_ is the shortest path length between nodes *i* and *j*^13^. To characterize anomalous random walks, we propose the concept of a MFTD *l*_*ij*_, which is the expected distance taken by a walker to first reach node *j* starting from node *i*. Intuitively, the traverse distance in a one-step jump is shorter for a walker when nodes are directly connected, while this distance tends to be larger for indirectly linked nodes. Inspired by the empirical findings that the lengths of links usually obey a power law distribution^17^, we adopt the power function 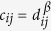 to describe the effective distance of one-step jump, where *β* named the cost exponent is a nonnegative value. In this situation, if the first step of the walk is to node *j*, the expected traverse distance required is 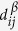; if it is to some other node *k*, the expected traverse distance becomes *l*_*kj*_ plus 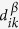 for the previous step already taken. Thus, we have


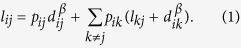


Using the Markov chains theory^18^,^19^, the MFTD *l*_*ij*_ of a anomalous random walk (see appendices) becomes


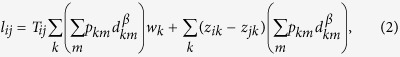


where *w*_*k*_ is the *k*th component of the stationary distribution of the anomalous random walk, *T*_*ij*_ is the MFPT from node *i* to node *j*, and *z*_*ij*_ is an element of the fundamental matrix *Z* = (*I* − *P* + *W*)^−1^. Specifically, when *β* = 0, the effective distances of one-step jumps are same (i.e., *c*_*ij*_ = 1). In this situation, it is easy to verify that the MFTD *l*_*ij*_ reduces to the MFPT *T*_*ij*_, which means that our paradigm can incorporate the commonly used MFPT as a special case. To further evaluate the search efficiency of an anomalous walker, we calculate the global MFTD 〈*L*〉 by averaging Eq. (2) over all pairs of source and target nodes, that is,


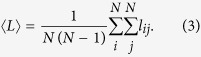


Substituting Eq. (2) into Eq. (3), we have


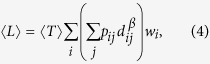


where 〈*T*〉 is the average of MFPTs over all pairs of nodes in the networks (see appendices). Here, 〈*L*〉 quantifies the ability of the anomalous walker to search and transport at the global scale on the network. In this context, smaller 〈*L*〉 represents a more effective way of achieving mobility. In the following we will demonstrate how these measurements can effectively characterize diverse anomalous random walks on networks.

### The MFTD scheme for characterizing Lévy walks

We first address a specific anomalous random walk — Lévy walks on networks. A Lévy walk exerts a power-law transition probability with the distance given by 
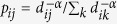
. Clearly, the tuning exponent *α* plays an important role in controlling the trade off between short-range and long-range jumping in one step, which in turn fully determines the behaviors of the Lévy walk. Specially, when *α* is very small, the walker visits all nodes with approximately equivalent probability. In contrast, the walker can only possibly hop to the nearest neighbors at an extremely large *α*. In this context, the Lévy walk degenerates to the generic random walk^2^. Using the balance condition, the stationary distribution of the Lévy walk can be expressed as


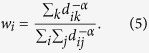


Inserting the above equation and the transition probability into Eq. (2) yields


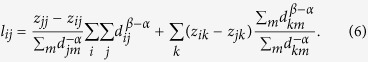


A similar calculation applied to Eq. (4), the global MFTD 〈*L*〉 of Lévy walks reads


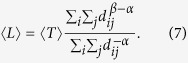


**Figure 1:**
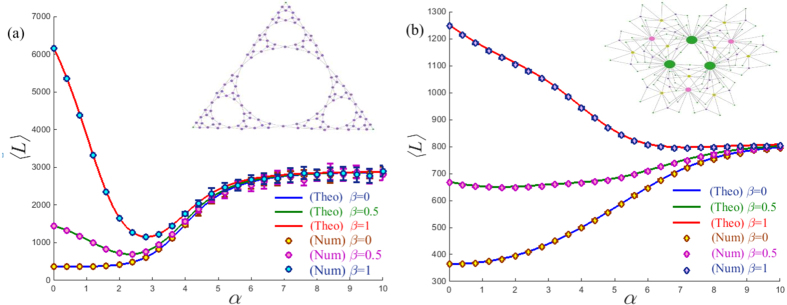
The global MFTD 〈*L*〉 as a function of *α*, for Lévy walks on (**a**) the planar Sierpiński gasket and (**b**) the (1, 2) flower model with the same size *N* = 366 nodes and *β* = 0, 0.5, 1, respectively. Symbols represent the values of 〈*L*〉 found numerically, while solid lines correspond to the theoretical prediction of Eq. (7). Error bars represent the mean first traverse distance 〈*L*〉 over 20 tests and each test is averaged over the ensemble of 50,000 independent runs.

To test the validity of Eq. (7), we report both the numerical and theoretical results of the global MFTD for Lévy walks taking place in the planar Sierpiński gasket^20^ and the (1, 2)-flower model^21^. These two networks are typical hierarchical nets having the same number of nodes and edges but exhibiting apparently distinct structural organizations, which can favor us to explore how the network structure directly influences the behavior of a Lévy walk. To achieve the numerical results, we compute the traverse distance required for a walker to travel from a source node to a target node chosen randomly and average over the ensemble of 50,000 independent runs for each test. Figure 1 shows an excellent agreement between numerics and Eq. (7) for the different cost exponents *β*. In particular, when *β* = 0, the minimum of 〈*L*〉 occurs at *α* = 0 regardless of the network structures, which reproduces the previous results based on the MFPT^22^. However, this result is unreasonable in practice without considering the distinct costs induced by the nearest-neighborhood jumps and the long-range hops. In contrast, we find that when *β* > 0, the profiles of different network organizations show clearly distinct behaviors. Specially, the profiles of the planar Sierpiński gasket display a clear minimum in the medium range *α*, which minimizes the search distance, (i.e., the global mean first traverse distance). However, such behavior is absent for the (1, 2)-flower model for *β* > 0, where they present a clearly monotonous tendency, see Fig. 1(b). Such difference can be intuitively explained when referring to their topological properties. Specifically, the Sierpiński gasket is a fractal network without the “small-world” property^20^, see its topological structure in Fig. 1(a). In contrast, the (1, 2)-flower network has the “small-world” feature and the “scale-free” characteristics^21^, as shown in Fig. 1(b). Meanwhile, we also notice that, when *α* is large, the transition probability of the Lévy walk degenerates to a generic random walk. Hence, all curves approach a fixed value for *α* > 9, see in Fig. 1(a) and (b), as expected.

**Figure 2:**
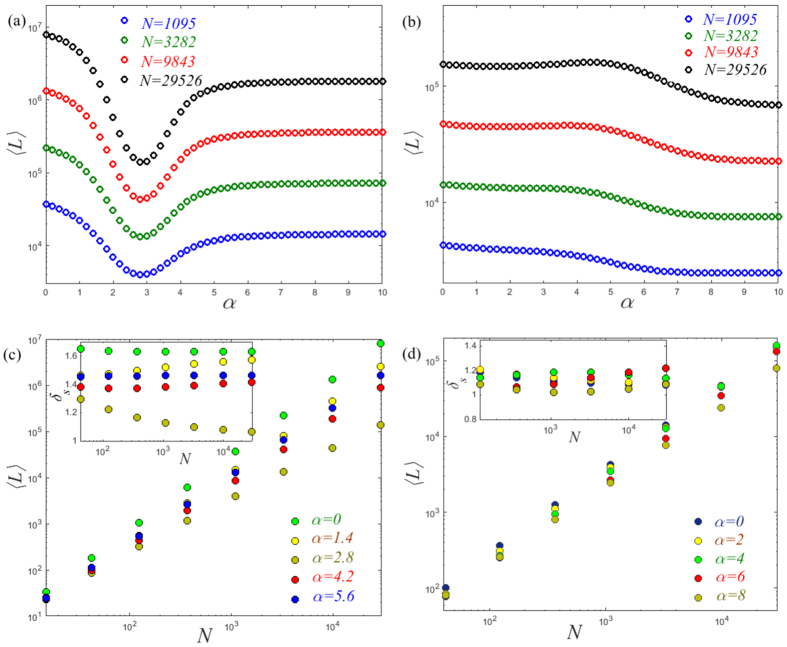
The global MFTD 〈*L*〉 as a function of *α* for Lévy walks on (**a**) the planar Sierpiński gasket and (**b**) the (1, 2) flower model over different network sizes *N*. The behaviors of 〈*L*〉 versus *N* for different exponents *α* on the planar Sierpiński gasket (**c**) and the (1, 2) flower model (**d**). In the insets, we show the plots of the successive slopes *δ*_*s*_ obtained from *ln*〈*L*〉 versus *lnN*. Note that here we set the cost exponent *β* = 1.

To further demonstrate the difference induced by network structure, we observe the size effect on the global MFTD 〈*L*〉 of the planar Sierpiński gasket and the (1, 2)-flower model. We find that the profiles of each network present the same tendency for different network sizes *N*, see Fig. 2(a) and (b). Interestingly, the result presented in Fig. 2(a) clearly shows the presence of a minimum 〈*L*〉 for different network sizes at the same exponent *α* = 2.8. The way in which 〈*L*〉 scales with network size *N* on the planar Sierpiński gasket seems to follow rather different behaviors depending on the tuning exponent *α*. Specially, when *α* ≠ 2.8, the global MFTD 〈*L*〉 follows a power law with network size *N*, see Fig. 2(c). This is supported by observing the almost invariant values of the successive slopes *δ*_*s*_ obtained from *ln*〈*L*〉 versus *lnN*, as shown in the inset of Fig. 2(c). Conversely, for *α* = 2.8, the successive slopes *δ*_*s*_ present a clearly decreasing tendency. However, for the (1, 2)-flower model, the 〈*L*〉 follows approximately a power law with network size *N*, see in Fig. 2(d). Note that here we choose the cost exponent *β* = 1 for convenience. However, such behavior of 〈*L*〉 versus *N* is general for an arbitrary cost exponent *β*.

**Figure 3:**
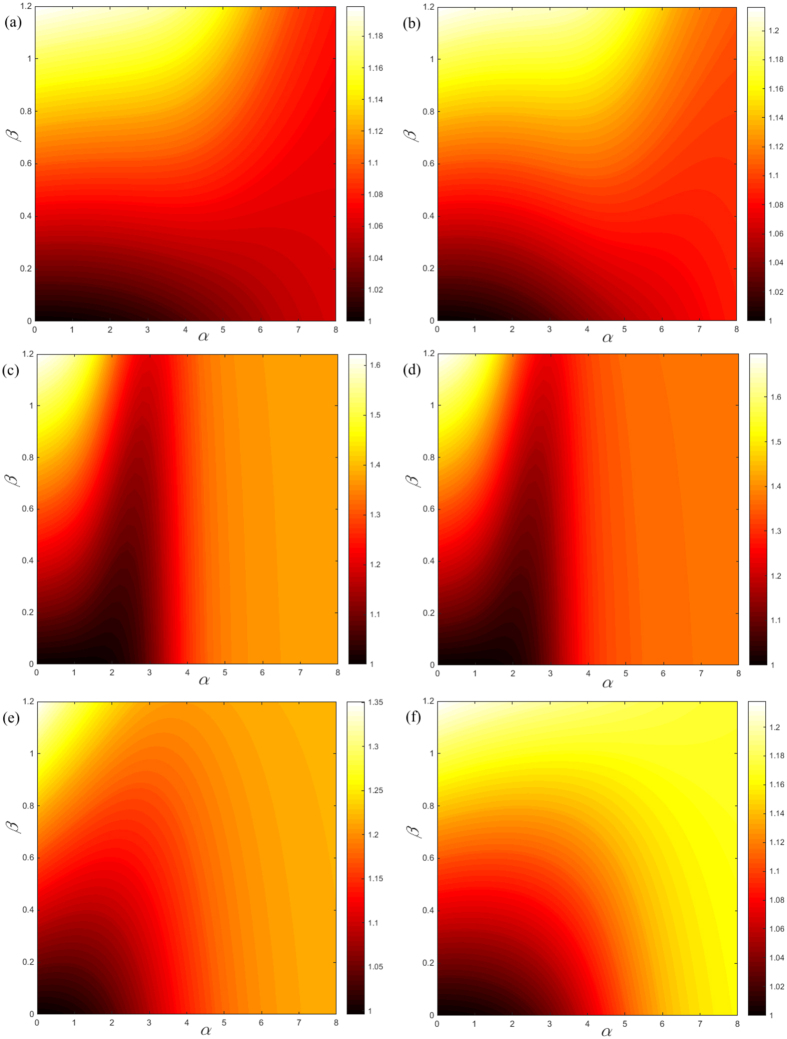
The measurement log_*N*_〈*L*〉 in the (*α*, *β*) parameter plane of (**a**) the BA model, (**b**) the (1, 2)-flower model, (**c**) planar Sierpiński gasket, (**d**) the (4, 5)-flower model, (**e**) the “Dolphin” network^24^, and (**f**) the e-mail network^25^.

Clearly, from Eq. (7), the cost exponent *β* plays an important role in controlling the search efficiency for Lévy walks. In order to explore how the optimal search efficiency of a Lévy walk changes with respect to the cost exponent *β*, we investigate the interplay between *β* and *α* for various networks including three synthetic models (the Barabási-Albert (BA) model^23^, the planar Sierpiński gasket^20^, and the (u, v)-flower model^21^) and two real networks (the “Dolphin” network^24^ and an e-mail network^25^). Here, for a fair comparison, we calculate the measurement log_*N*_〈*L*〉 in the (*α*, *β*) plane for eliminating the size effect of networks. Generally, regions with smaller log_*N*_〈*L*〉 indicate an efficient way of search and transport based on Lévy walks. Figure 3 shows contour maps of log_*N*_〈*L*〉 in the (*α*, *β*) plane computed for these selected networks. Interestingly, we find that distinct network structures lead to different patterns in the corresponding (*α*, *β*) plane. Specifically, the (*α*, *β*) planes generated from networks having the “small-world” characteristics (such as the BA model and the (1, 2)-flower model), demonstrate an “estuary” pattern — implying that Lévy walks are not the optimal way to search when *β* > 0.4. In contrast, typical fractal networks without the “small-world” property (for example, the planar Sierpiński gasket and the (4, 5)-flower model), result in a striking “flame” in the (*α*, *β*) planes, suggesting that there exists an optimal tuning exponent *α*, which minimizes the traverse distance for a broad range of cost exponents *β*. However, none of these patterns match the ones found in the Dolphin network and the e-mail network, whose (*α*, *β*) planes show “rippled” features, meaning that the optimal exponent *α* gradually increases with the cost exponent *β*. The (*α*, *β*) plane uncovers the relationship between network structure and the behavior of Lévy walks, which provides information to design more effective search strategies and transport mechanisms in different environments. Note that, for convenience, we choose the cost exponent in the range [0, 1.2] as this can highlight the effect of network structure on the transportation of the Lévy walks. Of course, the cost exponent can take other values outside this range. However, based on the Eq. (7), we find that the profiles of the Lévy walk present a clearly decreasing tendency independent of network structure for the large cost exponent *β*. In this situation, the Lévy walk is less efficient than the random walk for information diffusion and transportation. For this reason, we do not include the trivial results of the large cost exponents here.

Furthermore, we follow the spirit of the MFPT, and extract more statistics from the MFTD. Here, we introduce the average trapping distance (ATD) defined as follows:


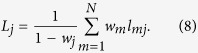


**Figure 4:**
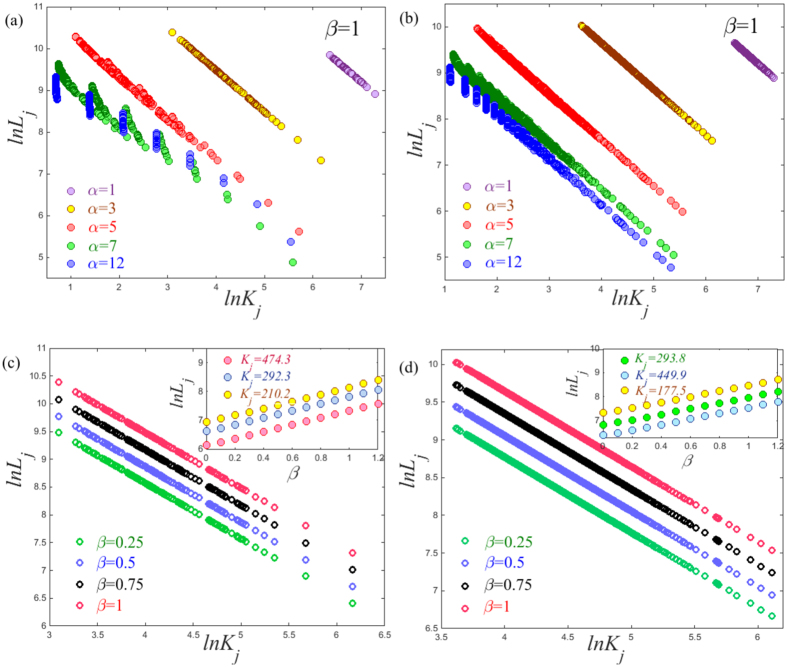
Plots of *lnL*_*j*_ versus *lnK*_*j*_ are presented for (**a**) the (1, 2)-flower model and (**b**) the BA model with network size *N* = 3282. The same plots with respect to different cost exponents *β* for (**c**) the (1, 2)-flower mode and (**d**) the BA model under the tuning exponent *α* = 3. In the inset, we show the plots of *lnL*_*j*_ versus *β* for different trapping nodes *j*. Note that here the values of *lnL*_*j*_ are calculated based on Eq. (8).

Clearly, the ATD *L*_*j*_ quantifies the mean of MFTD *l*_*mj*_ to the trap node *j* taken over all starting points with the stationary distribution. Submitting the results of Eqs (5) and (6) into Eq. (8) yields (see appendices)


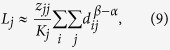


where 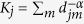 denotes the long-range degree of node *j*^13^ and the term *z*_*jj*_ is independent of the cost exponent *β*. Specifically, when *α* is small, the diagonal values of *Z* are almost the same. In this context, a clear scaling behavior emerges such that 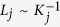 regardless of the underlying network structure. This is supported by observing the plots of *lnL*_*j*_ vs *lnK*_*j*_ shown in Fig. 4(a) and (b). With an increase of *α*, the slope of *lnL*_*j*_ versus *lnK*_*j*_ gradually decreases and finally asymptotically approaches to that of random walks as described in ref. 4. Results demonstrate the important role of *α* in shaping the ATD. Meanwhile, since the cost exponent *β* only influences the term 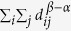 based on Eq. (9), changing the cost exponent *β* will just shift vertically the curve of *lnL*_*j*_ versus *lnK*_*j*_ but does not affect their profile. This is supported by observing the profiles of *lnL*_*j*_ versus *lnK*_*j*_, which present a similar tendency for different cost exponents *β* as illustrated in Fig. 4(c) and (d). We further find a linear relationship between *lnL*_*j*_ and *β*, when fixing the tapping position *j* and the tuning exponent *α*, see the insets in Fig. 4(c) and (d). The results are consistent with our theoretical prediction of the relationship *lnL*_*j*_~*Cβ*, where *C* is a constant value related to the fractal dimension of a given network (see appendices).

### The optimal condition of the PageRank search based on the MFTD theory

**Figure 5:**
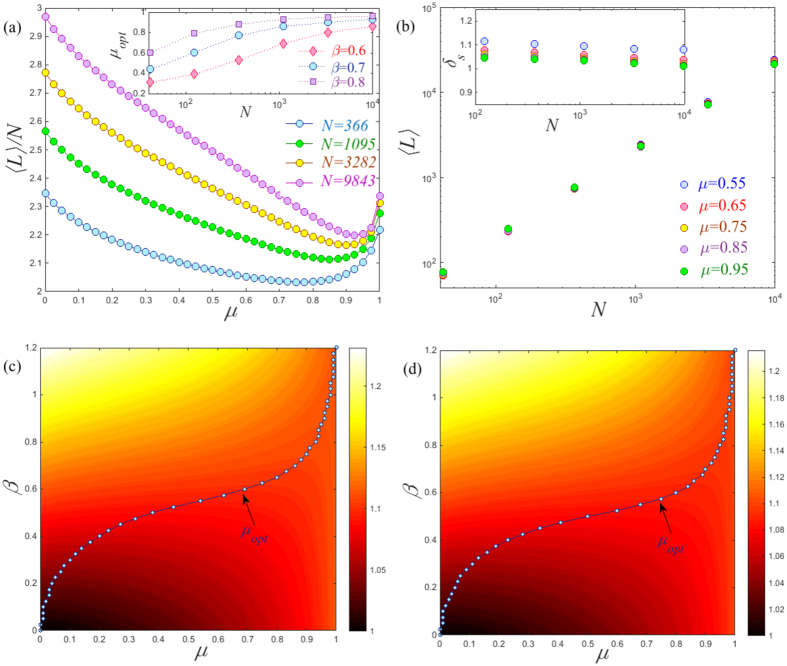
A comprehensive analysis of the PageRank algorithm on the (1, 2) flower model: (**a**) The global MFTD 〈*L*〉 as a function of *μ* over different network sizes *N*. In the inset, we show the plot of *μ*_opt_ versus *N* for different cost exponents *β*. (**b**) The behaviors of 〈*L*〉 versus *N* for different exponents *μ*, where we show the the successive slopes *δ*_*s*_ obtained from *ln*〈*L*〉 versus *lnN* in the inset. Note that here we set the cost exponent *β* = 0.7. The measurement log_*N*_〈*L*〉 in the (*μ*, *β*) parameter plane for (**c**) *N* = 1095 and (**d**) *N* = 3282, where the reverse “S” shaped lines point out the position of *μ*_opt_.

We finally apply the MFTD theory to characterize the famous PageRank search^16^. The PageRank search is widely used to compute the relevance of web pages. The transition probability *p*_*ij*_ of the PageRank search is


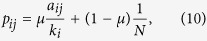


where 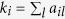 is the degree of node *i* and *μ* is the damping factor lying in the range [0, 1]. Clearly, the damping factor *μ* together with the network size *N* plays an important role in controlling the preference of visiting neighborhood or non-neighborhood nodes in one step, which in turn fully determines the behaviors of the PageRank search. To explore their effect on the behavior of the PageRank search, we study the global MFTD 〈*L*〉 for the PageRank algorithm on the (1, 2)-flower network. The (1, 2)-flower model is a hierarchical net having the “small-world” feature and the “scale-free” characteristics, which are the common features of various “web-page” networks^23^. The result presented in Fig. 5(a) clearly shows the presence of a minimum 〈*L*〉 for different network sizes. However, the value of *μ*_opt_, where the minimum 〈*L*〉 is achieved, increases gradually with the network size *N* as shown in the inset of Fig. 5(a). Such behavior is clearly distinct from that of a Lévy walk, where the optimal tuning exponent is independent of network size *N*. This unique behavior can be further tested by examining the form for 〈*L*〉 vs *N*, see Fig. 5(b). The way in which 〈*L*〉 scales with network size *N* seems to follow a power law behavior, which is supported by observing the almost invariant values of the successive slopes *δ*_*s*_ as shown in the inset of Fig. 5(b). These findings imply that the network size influences the optimal way of the PageRank search. Meanwhile, from the inset of Fig. 5(a), we also notice that the *μ*_opt_ relies on the cost exponent *β* and increases with increasing the cost exponent *β*. This characteristics shows more apparently in the (*μ*, *β*) planes as presented in Fig. 5(c) and (d), where the growth of *μ*_opt_ seems to follow the reverse “S” shaped line. This peculiar growth pattern means that the *μ*_opt_ increases slowly for a smaller *β* and then increases rapidly in the median range of *β*. Finally it will approach to *μ*_opt_ ≈ 1 for a large *β*. In this situation, the optimal PageRank search is the generic random walk. Of course, this is an extreme case seldom occurring in practice.

**Figure 6:**
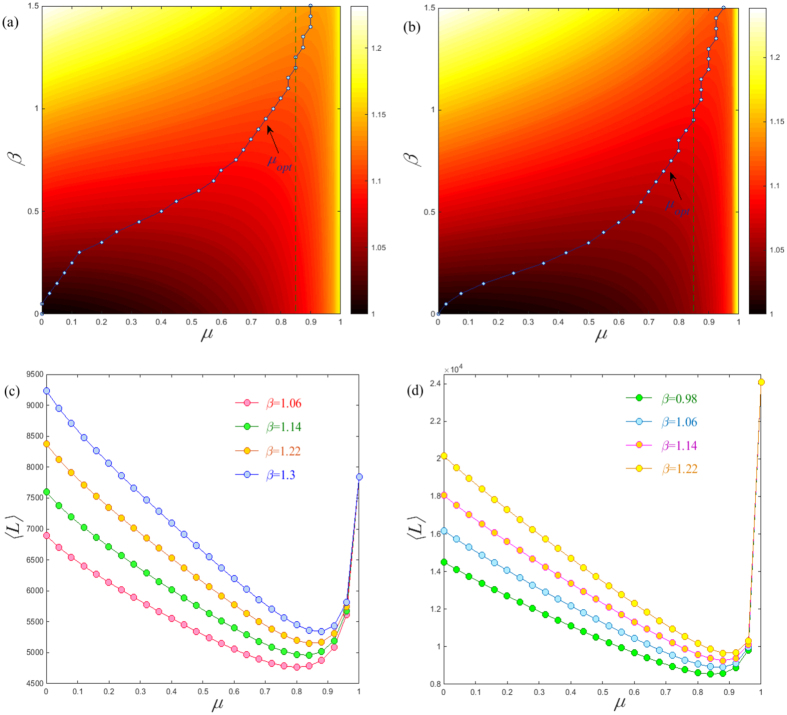
The measurement log_*N*_〈*L*〉 in the (*μ*, *β*) parameter plane of (**a**) web-Stanford^26^ and (**b**) Ego-Facebook^27^. The global MFTD 〈*L*〉 as a function of the damping factor *μ* on (**c**) web-Stanford and (**d**) Ego-Facebook with some special cost exponents for which *μ*_opt_ ≈ 0.85. Note that the web-Stanford network used here is a subgraph extracted from the original one for computation convenience with *N* = 2004.

Finally, we investigate the behavior for the PageRank search on two real networks (web-Stanford^26^ and Ego-Facebook^27^). The contour maps of the (*μ*, *β*) plane presented in Fig. 6(a) and (b) show a similar pattern, where the growth of *μ*_opt_ possibly follow the reverse “S” shaped line. Interestingly, we notice the existence of the median range of *β* for which an optimal search is achieved at the value of *μ*_opt_ ≈ 0.85. We highlight this special range in Fig. 6(c) and (d), where the global MFTD 〈*L*〉 is near its minimum value around *μ* ≈ 0.85. This is consistent with the ad hoc damping factor of the PageRank search which is suggested to be chosen around 0.85. Although we could not provide a complete explanation of *μ* ≈ 0.85 in practice, here we find a possible “reasonable” range of the cost exponent for the PageRank search, which may be further confirmed in the future by computing the topological distance versus the distance in the metric space constructed through the hyperbolic mapping of the Internet^28^. Moreover, we notice that the minimum 〈*L*〉 of the PageRank search is much smaller than that of generic random walks (i.e., *μ* = 1), which to some extent demonstrates the advantage of taking the PageRank search instead of generic random walks.

## Discussion

In summary, we have introduced the concept of the MFTD, a measure that takes into account of the cost of jumps in anomalous random walks, and which therefore is particularly suited to capture the interplay between the diffusion dynamics of anomalous random walks and underlying network structures. We obtain an exact expression for the MFTD and the global MFTD of anomalous random walks on complex networks. We show that our paradigm provides a unified scheme to characterize diffusion processes on networks, which incorporates the commonly used MFPT as a special case.

We demonstrate the effectiveness of these measures by applying them to Lévy walks and the PageRank search. Specially, we find that distinct network structures result in different patterns in the (*α*, *β*) planes, which explores the effect of the cost exponent *β* on behaviors of Lévy walks with respect to network structure. Moreover, we address how the tuning exponent *α* and the cost exponent *β* affect the trapping problem of Lévy walks. Specifically, we find that for a small value of *α*, the profiles present a uniformly linear scaling behavior regardless of network structure. Finally, when applied to the famous PageRank search, we explore the effect of network size and the cost exponent on the behavior of the PageRank search. In particular, we find that the growth of *μ*_opt_ seems to follow the reverse “S” shaped line with respect to the cost exponent. These findings will guide us how to design an optimal PageRank search in practice.

Our paradigm based on the MFTD is generic and can be applied to other anomalous random walks. However, the measurement of MFTD largely depends on an important factor — the cost exponent *β*. The obvious question we thus face is how to determine a “natural” value of the cost exponent *β* underlying networks. For an abstract network without any physical background, the cost exponent *β* can take any value in the interval [0, ∞). With respect to the efficiency of search and transport, we show the behaviors of anomalous random walks for some interesting ranges of the cost exponent *β* for convenience. In particular, we show the results of the cost exponent *β* lie in the interval [0, 1.2] for Lévy walks, while for the PageRank search, we present the cost exponent *β* lying in the range [0, 1.5]. For any real network, we believe that the cost exponent *β* can be estimated by computing the topological distance (i.e., the shortest path length) versus the distance in the metric space hidden behind an observable network as reported in refs 28, 29, 30. In this sense, as we believe, the cost exponent *β* is a physical parameter intrinsic to the real physical system, which links the topological distance and the distance in the hidden metric space.

Finally, to implement Lévy walks, we need to compute all shortest paths of a network which involves high computational costs especially for a large network. This issue has been already addressed in the previous literatures^13^,^14^ and is not the main scope of this paper. Nonetheless, we think one can use several excellent algorithms such as the preprocessing algorithm^31^, which is one possible solution for this problem. For the “natural” value of the cost exponent for the PageRank search, this can be achieved by computing the shortest path length versus the distance in the metric space which is constructed by a hyperbolic map of various web networks^28^. The cost exponent explores the relative relation between the topological distance of the web network and the distance in the hidden metric space. We do not expect the cost exponents underlying different web networks to be exactly the same. However, the cost exponent obtained in this way will help us to deepen our understanding of not only the PageRank search but also the other behaviors taking place on the Internet, for example the Internet routing.

## Additional Information

**How to cite this article**: Weng, T. *et al.* Navigation by anomalous random walks on complex networks. *Sci. Rep.* **6**, 37547; doi: 10.1038/srep37547 (2016).

**Publisher's note:** Springer Nature remains neutral with regard to jurisdictional claims in published maps and institutional affiliations.

## Supplementary Material

Supplementary Information
