# Supplementary material for: Navigation by anomalous random walks on complex networks
Source: Sci Rep. 2016 Nov 23;6:37547. doi: 10.1038/srep37547 (PMC5120342; doi:10.1038/srep37547)
Supplement: Supplementary Information [file srep37547-s1.pdf]

Navigation by anomalous random walks on complex networks

Tongfeng Weng, Jie Zhang, Moein Khajehnejad, Michael Small, Rui Zheng, and Pan Hui

The analytic expression of mean first traverse distance We follow the derivation of MFPT in Ref. [1] to calculate the MFTD on networks. We consider an arbitrary finite network consisting of N nodes. The connectivity is represented by the adjacency matrix A, whose entries $a_{ij} = 1$ (or 0) if there is (not) a link from nodes i to j. Let D denote the distance matrix with elements d_{ij} representing the shortest path length from node i to node j. In the process of anomalous random walks, at each step, the walker starting from node i arrives to node j with a non-zero transition probability p_{ij} regardless of the connectivity between nodes i and j. If the first step of the walk is to node j, the expected traverse distance required is d_{ij}^{β}; if it is to some other node k, the expected traverse distance becomes l_{kj} plus d_{ik}^{β} for the previous step already taken. Thus, we obtain

$$l_{ij} = p_{ij} d_{ij}^{\beta} + \sum_{k \neq j} p_{ik}(l_{kj} + d_{ik}^{\beta}), \tag{1}$$

where l_{ij} is the mean first traverse distance from node i to node j. Since $l_{jj} = 0$, Eq. (1) can be rewritten as

$$l_{ij} = \sum_{m} p_{im} d_{im}^{\beta} + \sum_{k} p_{ik} l_{kj}. \tag{2}$$

Let r_i denote the mean first return distance to node i starting from node i. In the same manner, r_i can be represented as

$$r_i = \sum_{k} p_{ik}(l_{ki} + d_{ik}^{\beta}). \tag{3}$$

Combining Eq. (2) and Eq. (3) together, we obtain the relation

$$(I - P)L = C - R, \tag{4}$$

where I denotes the identity matrix, and

$$L = \begin{pmatrix} l_{11} & l_{12} & \cdots & l_{1n} \\ l_{21} & l_{22} & \cdots & l_{2n} \\ \vdots & \vdots & \vdots & \vdots \\ l_{n1} & l_{2n} & \cdots & l_{nn} \end{pmatrix}, \tag{5}$$

$$C = \begin{pmatrix} \sum_k p_{1k} d_{1k}^{\beta} & \sum_k p_{1k} d_{1k}^{\beta} & \cdots & \sum_k p_{1k} d_{1k}^{\beta} \\ \sum_k p_{2k} d_{2k}^{\beta} & \sum_k p_{2k} d_{2k}^{\beta} & \cdots & \sum_k p_{2k} d_{2k}^{\beta} \\ \vdots & \vdots & \vdots & \vdots \\ \sum_k p_{Nk} d_{Nk}^{\beta} & \sum_k p_{Nk} d_{Nk}^{\beta} & \cdots & \sum_k p_{Nk} d_{Nk}^{\beta} \end{pmatrix}, \tag{6}$$

$$R = \begin{pmatrix} r_1 & 0 & \cdots & 0 \\ 0 & r_2 & \cdots & 0 \\ \vdots & \vdots & \vdots & \vdots \\ 0 & 0 & \cdots & r_n \end{pmatrix}. \tag{7}$$

Multiplying both sides of Eq. (4) by the matrix $W = \begin{pmatrix} w_1 & w_2 & \cdots & w_N \\ w_1 & w_2 & \cdots & w_N \\ \vdots & \vdots & \vdots & \vdots \\ w_1 & w_2 & \cdots & w_N \end{pmatrix}$ with the element w_i being the ith component of the stationary distribution, and using the fact that

$$W(I - P) = 0 \tag{8}$$

gives

$$WC - WR = 0. \tag{9}$$

From Eq. (9), the mean first return distance r_i reads

$$r_i = \frac{\sum_k \left(\sum_m p_{km} d_{km}^{\beta}\right) w_k}{w_i}. \tag{10}$$

Since the matrix $(I - P + W)$ has an inverse [1], we denote $Z = (I - P + W)^{-1}$. Multiplying both sides of Equation (4) by Z and using the fact that

$$I - W = Z(I - P) \tag{11}$$

gives

$$L = ZC - ZR + WL. \tag{12}$$

From the above equation, l_{ij} and l_{jj} can be expressed as

$$l_{ij} = \sum_k z_{ik} \left(\sum_m p_{km} d_{km}^{\beta}\right) - z_{ij} r_j + (wL)_j \tag{13}$$

and

$$l_{jj} = \sum_k z_{jk} \left(\sum_m p_{km} d_{km}^{\beta}\right) - z_{jj} r_j + (wL)_j. \tag{14}$$

Since $l_{jj} = 0$ and using Eq. (10), one has

$$l_{ij} = T_{ij} \sum_k \left(\sum_m p_{km} d_{km}^{\beta}\right) w_k + \sum_k (z_{ik} - z_{jk}) \left(\sum_m p_{km} d_{km}^{\beta}\right), \tag{15}$$

where $T_{ij} = \frac{z_{jj} - z_{ij}}{w_j}$ is the mean first passage time.

The analytic expression of global mean first traverse distance To further evaluate the search efficiency based on anomalous random walks, we introduce the global mean first traverse distance defined as

$$\langle L \rangle = \frac{1}{N(N-1)} \sum_i \sum_j l_{ij}. \tag{16}$$

Substituting Eq. (15) into Eq. (16), we obtain

$$\langle L \rangle = \langle T \rangle \sum_k \left(\sum_m p_{km} d_{km}^{\beta}\right) w_k + \frac{1}{N(N-1)} \sum_i \sum_j \sum_k (z_{ik} - z_{jk}) \left(\sum_m p_{km} d_{km}^{\beta}\right), \tag{17}$$

where $\langle T \rangle = \frac{1}{N(N-1)} \sum_i \sum_j T_{ij}$ is the global mean first passage time. Since column vectors of the matrix C are the same, the column vectors of the matrix ZC is also the same. Then, the last term of Eq. (17) will vanish due to

$$\sum \sum (ZC - (ZC)^T) = 0, \tag{18}$$

where the matrix $(ZC)^T$ represents the transpose of matrix ZC. So, the expression for $\langle L \rangle$ is reduced to

$$\langle L \rangle = \langle T \rangle \sum_k \left(\sum_m p_{km} d_{km}^{\beta}\right) w_k. \tag{19}$$

The analytic expression of average trapping distance for Lévy walks We now study the trapping problem for Lévy walks at an arbitrarily given node. Let L_j be the average trapping distance, which is the mean of MFTD L_{ij} to the trap node j, taken over the stationary distribution defined as follows:

$$L_j = \frac{1}{1-w_j}\sum_{i=1}^{N} w_i l_{ij}. \tag{20}$$

Substituting the expression of l_{ij} in the main text into Eq. (20) gives

$$L_j = \frac{1}{1-w_j}\sum_{i=1}^{N} w_i \left(\frac{z_{jj}-z_{ij}}{w_j}\frac{\sum_i\sum_j d_{ij}^{\beta-\alpha}}{\sum_i\sum_j d_{ij}^{-\alpha}}\right) + \frac{1}{1-w_j}\sum_{i=1}^{N} w_i \left(\sum_k (z_{ik}-z_{jk})\left(\frac{\sum_m d_{km}^{\beta-\alpha}}{\sum_m d_{km}^{-\alpha}}\right)\right). \tag{21}$$

Using the fact that $wZ = w$ [1] and with some calculation one obtains

$$L_j = \frac{1}{1-w_j}\frac{z_{jj}}{w_j}\frac{\sum_i\sum_j d_{ij}^{\beta-\alpha}}{\sum_i\sum_j d_{ij}^{-\alpha}} + \frac{1}{1-w_j}\sum_k z_{jk}\left(\frac{\sum_m d_{km}^{\beta-\alpha}}{\sum_m d_{km}^{-\alpha}}\right). \tag{22}$$

Empirically we find that the simulation values of the last term is far less than that of the first term and can be neglected in the analysis. In this context, Eq. (22) reduces to

$$L_j \approx \frac{z_{jj}}{K_j}\sum_i\sum_j d_{ij}^{\beta-\alpha}, \tag{23}$$

where $K_j = \sum_m d_{jm}^{-\alpha}$ named the long-range degree of node j [2]. Here, we omit the value w_j as it can be approximated as zero when the network size N is very large. Moreover, for the fractal network with the fractal dimension d_f, the network diameter M can be approximated as $M \sim N^{\frac{1}{d_f}}$. Approximating M as a continuous variable, the term $\sum_i\sum_j d_{ij}^{\beta-\alpha}$ scales as [3]

$$\sum_i\sum_j d_{ij}^{\beta-\alpha} \sim N\int_1^M x^{\beta-\alpha}x^{d_f-1}dx \sim \begin{cases} N\frac{N^{\frac{d_f+\beta-\alpha}{d_f}}-1}{\beta+d_f-\alpha}, & \alpha \neq d_f+\beta \\ \frac{NlnN}{d_f}, & \alpha = d_f+\beta \end{cases}. \tag{24}$$

Plugging Eq. (24) into Eq. (23), we have a linear relationship between lnL_j and β (i.e., $lnL_j \sim C\beta$ where C is a constant value determined by the fractal dimension d_f), when the position of the trapping node j and the tuning exponent α are fixed.

[1] Grinstead, C. M. & Snell, J. L. Introduction to Probability (American Mathematical Society, 2006).
[2] Riascos, A. P. & Mateos, J. L. Long-range navigation on complex networks using lévy random walks. *Phys. Rev. E* **86**, 056110 (2012).
[3] Li, G. *et al.* Optimal transport exponent in spatially embedding networks. *Phys. Rev. E* **87**, 042810 (2013).
